# Factors Associated with Reproductive Success in Captive Vancouver Island Marmots (*Marmota vancouverensis*)

**DOI:** 10.3390/ani14030387

**Published:** 2024-01-25

**Authors:** Laura H. Graham, Emily M. Leishman, Kahlee Demers, Douglas P. Whiteside, Malcolm McAdie

**Affiliations:** 1 WRG Conservation Foundation, West Montrose, ON N0B 2V0, Canada; 2 College of New Caledonia, Prince George, BC V2N 1P8, Canada; 3 Department of Animal Biosciences, University of Guelph, Guelph, ON N1G 2W1, Canada; eleishma@uoguelph.ca; 4 Independent Researcher, Maple Ridge, BC V2W 0A9, Canada; 5 Wilder Institute/Calgary Zoo, Calgary, AB T2E 7V6, Canada; dougw@calgaryzoo.com; 6 Marmot Recovery Foundation, Nanaimo, BC V9R 6X6, Canada; malcolmmcadie@shaw.ca

**Keywords:** Vancouver Island marmot, endangered species, reproduction, stress

## Abstract

**Simple Summary:**

The Vancouver Island marmot (*Marmota vancouverensis*) is Canada’s most endangered endemic mammal and is found exclusively on Vancouver Island in British Columbia. In the 1990s, the wild population drastically declined to fewer than 100 animals, primarily due to habitat alterations. In 1997, a conservation breeding-for-release program was established to supplement wild marmot populations as part of the National Recovery Plan. Retrospective analyses of captive breeding studbook records since 2000 indicate that among animals of proven fertility, the proportion of breeding pairs weaning a litter of pups is only 53%, which is less than optimum to support the reintroduction of animals to increase the wild population. Factors associated with the female were found to have the greatest impact on the odds of successfully weaning a litter, including age of the female and her previous success, although the age of the male also had an effect. A comparison of adrenal function between new and established breeding pairs, and successful and unsuccessful breeding pairs, indicated lower fecal glucocorticoid metabolite concentrations in established and successful pairs. This suggests that certain pairings are associated with lower stress than others, possibly reflecting social buffering of the stress response. The results of these analyses will be used to inform the captive management breeding program as it continues to supplement the wild populations of this endangered species.

**Abstract:**

The Vancouver Island marmot (*Marmota vancouverensis*) is Canada’s most endangered endemic mammal. In 1997, a conservation breeding-for-release program was established to supplement wild marmot populations. Retrospective analyses of captive breeding studbook records since 2000 indicate the age of the sire and the dam significantly impacted the odds of successfully weaning a litter. Dams and sires between 5 and 7 years of age had more than double the odds of reproductive success compared to older animals. Successful reproduction by the dam in the previous year also doubled the odds of successfully weaning a litter in subsequent years. Assessment of adrenal function via fecal glucocorticoid analyses indicated established breeding pairs had decreased stress compared to new pairs (5.74 ± 0.28 ng/g vs. 7.60 ± 0.34 ng/g; *p* < 0.0001). Pairs that were ultimately successful at weaning pups in a breeding season had decreased stress compared to unsuccessful pairs (6.05 ± 0.34 ng/g vs. 7.22 ± 0.28 ng/g; *p* = 0.0006). These endocrine results suggest social buffering via familiarity and breeding/pair bond formation may be decreasing stress in established and successful pairs, respectively. The results of this study will be used to assist in the captive breeding management of this species to optimise numbers of animals produced to supplement the wild populations.

## 1. Introduction

The Vancouver Island marmot (*Marmota vancouverensis*) is a large fossorial rodent endemic to Vancouver Island, British Columbia, Canada [1], and is classified as Critically Endangered by the International Union for Conservation of Nature (IUCN) Red List of Endangered Species [2]. Its preferred habitat is subalpine meadows that are maintained as meadows by natural factors such as avalanches, snow-creep, and fire. It is a true hibernator, entering hibernation in early fall and emerging from hibernation in late April. While the Vancouver Island marmot was poorly studied prior to its population decline, observations suggest it typically lives in small colonies (fewer than five adult animals), with dispersal of young animals to other colonies in a metapopulation lifestyle. Reproductive colonies include one or more family groups, with each family group consisting of an adult male, usually one but sometimes two adult females, two-year-old pups, and young of the year [3]. Marmots breed shortly after emergence from hibernation, have a gestation of ~32 days and wean their young within approximately 28 days, typically in July. Some females are reproductively mature at age two, but most do not breed until age three or four, producing litters of three to four pups every other year. Both sexes disperse, typically at age two. The maximum observed age is 12 years in the wild and 14 years in captivity [1]. Like other alpine-dwelling marmot species, the Vancouver Island Marmot has low reproductive output and the small population size makes it more susceptible to disease and stochastic demographic or weather events. There is evidence that the Vancouver Island marmot is limited at low population density by Allee effects. Brashares et al. [4] reported that at low densities, marmots had larger home-ranges, less interaction with conspecifics, spent more time in antipredator vigilance, and less time on feeding. They also had lower per capita survival and reproduction. Additional limitations associated with low populations include genetic isolation and limited habitat within the dispersal distance of occupied colonies [1].

In the 1990s it was observed that clear-cut logging practices were creating temporary artificial colony habitats attracting young dispersing animals [5]. However, the net reproductive value of colonies in clearcut habitats was less than half that of natural colonies. It was suggested that recently logged habitats were acting as demographic “sinks” by consuming more dispersers than they produced, and therefore impeding replacement in existing colonies and recolonization of distant natural habitats. The species had been listed as endangered under Canada’s Species at Risk Act in 1978, and by the mid-1990s the wild population had decreased precipitously to approximately 70 animals, and to only 30 animals by 2003 [1]. The proximate cause of the decline was observed to be increased predation of colonies established in clearcut habitats compared to colonies in natural habitats, with the ultimate cause being the alteration of the ecosystem by resource extraction. The most common predator of marmots in alpine meadows is the golden eagle. However, clearcut habitats not only attract dispersing marmots but also Cervidae species which, in turn, draw larger predators like cougars and wolves that opportunistically prey on marmots.

The current National Recovery Strategy for the Vancouver Island marmot has the goal of establishing two geographically distinct, wild meta-populations having a greater than 90% probability of persisting for over 100 years, without augmentation from the captive program [6]. In 1997, a conservation breeding-for-release program was established to supplement wild marmot populations as part of the National Recovery Plan [7]. There were originally four institutions participating in the captive breeding program, including Toronto Zoo, followed by Calgary Zoo and a private facility in BC, and ultimately a purpose-built facility on Vancouver Island: the Tony Barrett Mt Washington Marmot Recovery Centre, in 2001 [8]. These institutions and associated professionals work together as the Vancouver Island Marmot Captive Breeding Group to facilitate the goals of the National Recovery Plan for the species.

As of 2023 there have been 630 captive marmots released to the wild over the last 20 years, with ~30 animals released annually [9]. The combination of reproduction in the wild (by wild and captive-born marmots) and supplementation of numbers by captive releases led to a dramatic increase in the population of marmots in the wild to almost 375 animals in 2012/2013 [10]. However, the reduction in captive animal releases from 2015–2017 corresponded with a decline in the wild population due to stochastic weather and predation events, at which point the captive breeding program and reintroductions were revitalized. The continued supplementation of the wild population by captive-bred individuals is still essential to prevent the extinction of Vancouver Island marmot in the wild due to stochastic events [10]. The current wild population is estimated at 309 [9].

Vancouver Island marmots under human care are managed according to the principles of small population management (the small-population paradigm), with the emphasis on managing genotypes to minimize the rate of genetic decay [11,12]. Breeding pairs are chosen using mean kinship values to maximize genetic diversity, and inbreeding coefficients to avoid inbreeding. However, a recent paper has suggested that reproductive viability analyses (RVA) can be an additional tool to make evidence-based decisions about pairing animals for breeding endangered species in captivity [13]. RVA is a tool that analyzes the inherent biological and reproductive characteristics of individual animals (e.g., age, parity, and rearing type) and breeding pairs (e.g., experience as a pair and age difference) that impact the likelihood of successful reproduction. The authors suggested that analyzing past breeding recommendations and their results can produce timely results from which evidenced-based management could be made to improve small population management of captive endangered species. The objective of the current study was to retrospectively analyze management, life history, and physiological variables associated with breeding success in captive Vancouver Island marmots from 2000–2021. The results of these analyses will be used to inform the captive management breeding program as it goes forward.

## 2. Materials and Methods

### 2.1. Animals and Studbook Data Analyses

Vancouver Island marmots were housed at four captive breeding facilities between 2000 and 2021: Toronto Zoo, Calgary Zoo, Mountain View Conservation and Breeding Centre, and the Tony Barrett Mt Washington Marmot Recovery Centre. All animals were managed collaboratively among the captive breeding facilities via a common protocol developed by the Captive Management Group and described by Aymen et al. [14]. Data on breeding pair success were obtained from the Vancouver Island marmot studbook [12] from the first year of successful breeding in captivity (2000) to 2021. Only pairs where both the male and female were of proven fertility at least once between 2000 and 2021 were included in the statistical analyses (N = 379 pairs, N = 80 females, and N = 93 males). Breeding pairs were classified as established if they had been paired for breeding in previous years, and as new if they had never previously been paired together for breeding. New pairs were introduced just prior to or during hibernation and had been together <1 year, while established pairs had been housed together for ≥1 year.

### 2.2. Fecal Sample Collection and Hormone Extraction for Endocrine Analyses

Fecal samples were collected at least 3 times per week from the enclosures of breeding pairs (N = 17 pairs) housed at Toronto Zoo and Calgary Zoo for the first three weeks following emergence from hibernation. Samples were frozen at −20 °C within 24 h of defecation and stored until endocrine analyses. Glucocorticoid metabolites were extracted from feces using established protocols [15]. In brief, glucocorticoid metabolites were extracted from an aliquot (0.48–0.52 g) of each sample via agitating in 5.0 mL of 80% aqueous MeOH overnight on an orbital shaker (Junior Orbit Shaker, Lab-Line Instruments Inc., Melrose Park, IL, USA). Following agitation, the extract containing cortisol metabolites was separated from the feces via centrifugation (25 min at 2500 rpm; Beckman Model J-6M, Brea, CA, USA), and stored in evaporation-proof vials (2 mL vials with an O-ring screw cap) at −20 °C until enzyme-immunoassay.

### 2.3. Enzyme-Immunoassay (EIA)

Fecal extracts were diluted (1:4 to 1:32) in Trizma assay buffer (0.02 M Trizma, 0.300 M NaCl, 0.1% BSA; pH 7.5) prior to assay. Fecal glucocorticoid metabolite (FGM) concentrations were quantified using a cortisol EIA with cortisol (18–250 pg/50 μL) in the standard curve, in a similar assay procedure as described for polar bears but with a different cortisol metabolite antibody and label [16]. In brief, microtitre plates were coated with affinity-purified goat anti-rabbit gamma globulin (25 μg/plate) dissolved in coating buffer (0.015 M Na_2_CO_3_, 0.035 M NaHCO_3_; pH 9.6), and incubated overnight at room temperature. Wells were emptied and refilled with Trizma assay buffer and stored at room temperature for at least 30 min prior to use to block non-specific binding. Coated plates were washed (0.04% Tween 20) and 50 μL of diluted sample and standards were dispensed. Horse-radish peroxidase-labeled cortisol was dispensed, followed by anti-cortisol antibody (Antibody #R4866; CJM Munro, UC Davis). Plates were incubated overnight at 4 °C. Plates were then washed and 200 μL of substrate solution (0.5 mL of 0.016 M tetramethylbenzidine in dimethylsulphoxide, and 100 mL of 0.175 M H_2_O_2_ diluted in 24 mL of 0.01 M C_2_H_3_O_2_Na; pH 5.0) was added to each well. After incubation (45 min, room temperature) the enzyme reaction was stopped with 50 μL of stop solution in each well (3 M H_2_SO_4_). The optical density was measured at 450 nm (reference 595 nm). If sample duplicates had a percent coefficient of variation (CV) greater than 10%, samples were reanalyzed. A low and high control was assayed with each plate and the interassay CV was <15% for each. All chemicals and laboratory consumables were purchased from either Millipore Sigma (Oakville, ON, Canada) or Thermo Fisher Scientific (Mississauga, ON, Canada), unless otherwise stated. Final FGM concentrations were reported in the units of ng/g feces.

### 2.4. Assay Validation

As an analytical validation, pools of fecal extracts from both male and female Vancouver Island marmots were combined and assayed with the cortisol EIA. To establish parallelism, serial two-fold dilutions of each sample pool were tested for comparison displacement curves. Recovery of exogenous hormone was measured by spiking a baseline diluted sample pool with cortisol ranging from 7.8 to 125 pg/well. The average percent recovery was calculated by dividing the measured concentration of hormone by the expected concentration of hormone multiplied by 100, and was 96.3%.

As a physiological validation, fecal samples were assayed after a known stressful event in two marmots to ensure that the cortisol enzyme-immunoassay was capable of measuring the increase in fecal glucocorticoid metabolites associated with an increase in adrenal activity. The known stressful events included relocation to a new enclosure and exposure to models of predators for assessment of predator-recognition behavior.

### 2.5. Calculating ‘Species Baseline’ of FGM in Vancouver Island Marmots

Fecal glucocorticoid metabolite concentrations were quantified in 1499 samples collected (opportunistically or for other studies) from 102 Vancouver Island marmots (wild and captive). An approximate ‘species baseline’ was calculated from this data set using an iterative process similar to that used for the calculation of individual animals’ baseline [17]. This ‘species baseline’ was used to give context to the FGM concentrations quantified in the breeding pairs in the present study, as well as the data supplied for the physiological validation of the enzyme-immunoassay.

### 2.6. Statistical Analyses

Studbook data was used to evaluate factors influencing the likelihood of reproductive success. A linear model with a binary distribution was used to model the probability that litters were successfully weaned. Univariable analysis of all possible fixed effects was first conducted. These independent variables included: the age of the sire and dam, the age class difference between the sire and dam, whether the sire or dam had any prior reproductive success, whether the dam was successful in the previous year, whether the sire or dam were previously transferred, whether the sire and dam were wild or captive-bred, whether both parents were wild or captive-bred, and whether it was an unfamiliar or familiar pair. The multivariable model was then selected including only factors with a *p* < 0.1 (tendency) at the univariable level. Results of the univariable and multivariable models are presented as odds ratios (OR) with associated 95% confidence intervals (CI).

To evaluate factors influencing FGM concentrations in breeding pairs, a generalized linear mixed model with a lognormal distribution was used with the dependent variable FGM concentration (ng/g), the dependent variables reproductive success (yes vs. no), and pairing type (new vs. established), corrected for institution (Calgary Zoo and Toronto Zoo). The interaction between reproductive success and pairing type was also included in the model. A repeated statement was included to account for repeated pairs over the years. Reproductive success (yes) was defined as weaned pups. If pups were not produced or successfully weaned, then the occurrence was designated as unsuccessful (no). Familiar pairs were defined as pairs who had been together for at least 1 previous breeding season. Results from this model are presented as back-transformed least-square means (LSmeans) ± the standard error (SE).

All analyses were conducted using SAS Studio (version 9.4., SAS Institute Inc., Cary, NC, USA). The alpha level for determination was 0.05 and tendencies reported between 0.05 and 0.1. *p*-values for comparisons were adjusted using Tukey’s HSD.

## 3. Results

### 3.1. Studbook Data Analyses

#### 3.1.1. Univariable Analysis

Four variables were identified at the univariable level with *p* < 0.1 (Table 1). These variables included the age class of the sire (*p* = 0.0059), age class of the dam (*p* = 0.0114), the success of the dam in the previous year (*p* = 0.0034), and whether there was a difference in age class between the sire and dam (*p* = 0.0702). These variables proceeded to the multivariable analysis. 

#### 3.1.2. Multivariable Analysis

The initial multivariable model contained the four variables with *p* < 0.1 at the univariable level. In this model, the effect of sire age (*p* = 0.0163), dam age (*p* = 0.0971), and dam success in previous year (*p* = 0.0015), remained under the *p* < 0.1 threshold; however age class difference did not (*p* = 0.2919). Therefore, the model was rerun excluding the age class difference variable and the results of the revised multivariable model (including three variables) are presented. 

Sire age significantly influenced the odds of successful litters (*p* = 0.0134). Compared to the oldest sires (group C: 8–14 y), sires in group B (5–7 y) had 2.3x higher odds of having successful litters. The youngest sires (group A: 2–4 y) were also 2.4x more likely to have successful litters compared to the oldest sires. There was no difference in the odds of successful litters between groups A and B (OR_B vs. A_ = 0.933; 95%CI: 0.556–1.565). 

Dam age significantly influenced the odds of successful litters (*p* = 0.0451). Compared to the oldest dams (group C: 8–14 y), dams in group B (5–7 y) had 2.1x higher odds of having successful litters (95%CI: 1.166–3.360). The odds of success between the youngest dams (group A: 2–4 y) and oldest dams were not different (OR = 1.476; 95%CI: 0.830–2.624). Additionally, there was no difference in the odds of successful litters between groups A and B (OR_B vs. A_ = 1.394; 95%CI: 0.792–2.453).

Lastly, whether the dam produced a successful litter in the previous year influenced the odds of a successful litter (*p* = 0.0013). Dams who were successful in the previous year were 2.2× more likely to have a successful litter in the current year compared to dams who were unsuccessful in the previous year (95%CI: 1.353–3.461).

### 3.2. Endocrine Analyses

**Figure 1:**
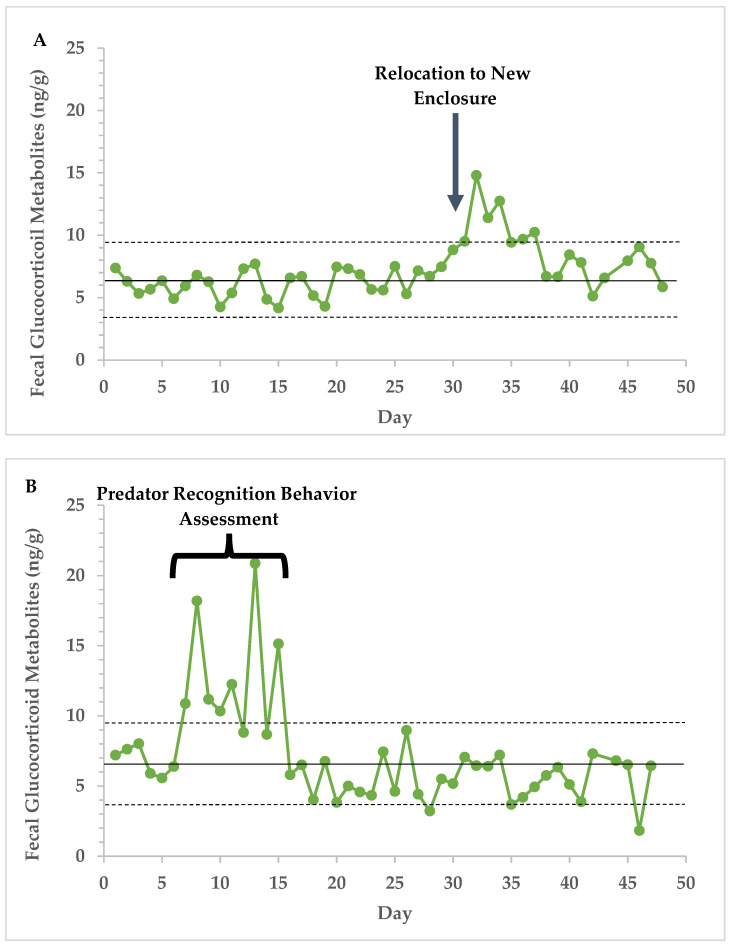
FGM concentrations (ng/g) in two individual marmots exposed to housing disruptions. One marmot was moved to new enclosure (**A**), and one marmot was repeatedly exposed (N = 4 incidences) to models of predators for assessment of predator recognition behavior (**B**). For context, solid and dashed lines represent calculated species baseline FGM concentration and standard deviation, respectively (6.59 ± 3.10 ng/g).

Elevations in FGM concentrations were associated with disruptions in housing, such as relocation to a new enclosure (Figure 1A), and being exposed to models of predators to assess predator-recognition behavior (Figure 1B). This suggests that the enzyme-immunoassay was measuring FGM concentrations reflective of adrenal function.

**Figure 2:**
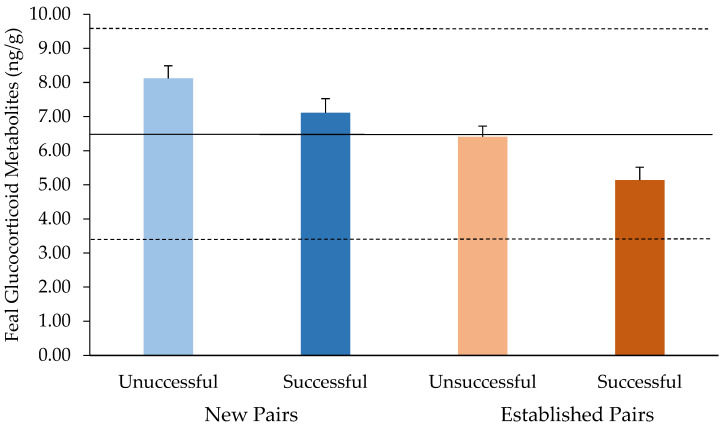
FGM concentrations (ng/g) of different pairing types (New vs. Established) and weaned litter success. Data is presented as back-transformed LSmeans ± SEM. Main effects of pair type and reproductive success on FGM concentrations were significant (*p* < 0.001) with no interaction. For context, solid and dashed lines represent calculated species baseline FGM concentration and standard deviation, respectively (6.59 ± 3.10 ng/g).

There was a significant difference between FGM concentrations of successful and unsuccessful breeding pairs (*p* = 0.0006). Pairs who successfully weaned pups had lower FGM concentrations (6.05 ± 0.34 ng/g) compared to unsuccessful pairs (7.22 ± 0.28 ng/g). There was a significant difference in the FGM concentrations in different pair types (*p* < 0.0001). FGM concentrations were lower for established pairs (5.74 ± 0.28 ng/g) compared to new pairs (7.60 ± 0.34 ng/g) (Figure 2). The interaction between reproductive success and pairing type on FGM concentrations was not significant (*p* = 0.3194).

## 4. Discussion

The Vancouver Island marmot has been managed under a National Recovery Strategy since 1994 [6]. An important component of the recovery strategy is the captive breeding/release program. Since the first marmot was bred in captivity in 2000, there have been 759 marmots born in captivity and 630 marmots released to the wild as of 2023. Despite this success, an average of only 39% of annual captive pairings produce a weaned litter [9]. Even when eliminating animals that have never bred in captivity, as we did in our analyses, the success rate was only 53.0% (201 litters/389 pairings). A population and habitat viability analyses in 2015 [10] suggested that the current wild population needs augmentation of captive born marmots annually to prevent extinction. To meet this goal, it is necessary to understand various factors that impact the odds of a breeding pair producing a weaned litter. With this knowledge, recovery program managers can maximize existing infrastructure and breeding efficiency, and also breed captive marmots in a more predictable fashion to ensure a better preservation of existing genetics and maintain long-term demographic and genetic integrity.

An earlier study on Vancouver Island marmots in the conservation breeding program looked at factors leading to successful reproduction [18]. The authors reported that age of the dam influenced the likelihood of successful reproduction, with females between 5 and 7 years of age having greater success than females younger or older. A similar effect of female age on reproductive output was reported earlier in a combined data set of wild and captive Vancouver Island marmots, with reproduction highest in females of at least 3 years of age [19]. The present study, which included a much larger number of animals all of which had proven fertility, confirmed this conclusion. Our results indicate that females between 5 and 7 years of age had more than double the chance of successful reproduction than older females (2.1×; 95%CI: 1.166–3.360), with the odds of younger females successfully reproducing no different from the two older age classes. Similarly, the relative importance of female age on reproductive success has been reported after multi-factor analyses of studbook information (RVA) in other endangered species in captivity, such as fennec foxes [11]. Age was the only male factor that impacted the odds of reproductive success, with marmots less than 8 years of age having more than double the odds of successfully weaning a litter than males ≥ 8 years of age (age group A 2.4×; Age Group B: 2.3×). Earlier studies [18,19] had too few animals to provide information on the effect of sire age on the odds of reproductive success in captive Vancouver Island marmots. Long-term studies in wild yellow-bellied marmots indicate that reproductive success peaks at ~7 years of age in both sexes and declines afterwards [20]. However, the reasons for the reproductive senescence may be different between the two sexes, with physiological variables implicated in females while in male yellow-bellied marmots, increased reproductive costs due to competition likely directs the loss of reproductive fitness, an obstacle that captive male Vancouver Island marmots are not subjected to.

In our initial univariable analyses, we included both reproduction in any previous year and reproduction in the previous year, but only reproduction in the previous year had a significant impact by nearly doubling the odds of successful breeding (1.871×; 95%CI: 1.233–2.84). This was a somewhat surprising result, though it was also reported in the earlier study by Casimir et al. [18]. In their study, the production of young in any previous year tended (*p* < 0.07) to increase the odds of successful reproduction in successive years. In our final multivariable model, weaning a litter the previous year more than doubled the odds of reproductive success in the subsequent year (2.2×; 95%CI: 1.353–3.461; *p* = 0.0013). This is contradictory to what is observed in wild Vancouver Island marmots which have a between-litter interval of 1.86 years, significantly longer than that observed in their captive counterparts in an early comparison of reproductive traits of wild and captive marmots [19]. Bryant [19] suggested that improved body condition could account for the shorter between-litter interval in marmots in human care. Our results indicate that in female marmots in human care, successful reproduction in a breeding season does not impair reproduction in the following breeding season as observed in wild marmots, but instead actually increases the odds of successful reproduction the following year. Vancouver Island marmots under human care are housed in low temperatures (5–7 °C) in the fall to encourage hibernation, but are not normally exposed to the extremely low temperatures that wild marmots are exposed to. There are also other benefits to life under human care including parasite control and a consistently nutritious diet. These conditions would enable captive females to arouse from hibernation in better body condition at the beginning of the breeding season compared to wild marmots. A study on wild alpine marmots indicated that reproduction depleted fat reserves and only those females that had good body condition reproduced successfully [21]. Studies indicate that in alpine marmots the annual energetic demands in females of reproducing, lactating, and hibernating with pups exceeded the amount of body fat that could be accumulated in one summer, thus reducing the likelihood of reproducing every year [22,23]. 

Although we found no impact of pair type (established vs. new) on the odds of reproducing successfully in captivity in a breeding season, our data does provide some hints that familiarity can be a bonus to overall reproductive output. Of the litters weaned in successive years (N = 91 litters), 84.6% were in established pairs where the pair had been together for at least 1 year previously. Casimir et al. [18] found that both male and female Vancouver Island marmots had greater odds of producing a litter if they had been housed together for at least 1 year prior to the breeding season and suggested that familiarity may play a role in reproductive success in Vancouver Island marmot. 

When we compared FGM between successful and unsuccessful pairs, unsuccessful pairs had significantly higher FGM concentrations compared to successful pairs (7.22 ± 0.28 vs. 6.05 ± 0.34 ng/g; *p* = 0.0006). An obvious interpretation would be that the unsuccessful pairs were highly stressed and high glucocorticoids were suppressing fertility. This effect of stress on fertility, including high glucocorticoid concentrations, is well established in many mammalian species [24,25]. However, the FGM concentrations observed in the unsuccessful pairs were less than one standard deviation from the ‘species baseline’. The Reactive Scope Model provides a theoretical context to understand how animals respond to stress, including the role of glucocorticoids [26]. In this model, increases in physiological variables associated with stress, such as glucocorticoids, that are within the reactive scope of a species, are adaptive, allowing an animal to adjust its physiology to deal with the ‘normal’ stresses of life. It is only when concentrations increase into the range of homeostatic overload that the negative effects of stress on reproduction become apparent, with high glucocorticoids becoming disease-causing. Although the Reactive Scope Model is only theoretical and has not been applied empirically to marmots [27], it seems reasonable to assume that concentrations of FGM indicative of homeostatic overload would be more than one standard deviation from the calculated ‘species baseline’. An alternative explanation is that the increased FGM concentrations observed in unsuccessful pairs are not the cause of reproductive failure but rather the result of an unknown factor, possibly related to reproduction. Studies on wild yellow-bellied marmots (*Marmota flaviventris*) found no difference in FGM concentrations between successful and unsuccessful pairs but yellow-bellied marmots have a different, more polygynous social system than Vancouver Island marmots [28]. Furthermore, wild individuals of both yellow-bellied marmots and Vancouver Island marmots have physiological variables indicating they are more highly stressed than captive individuals, suggesting that the natural environment in which these animals occur is generally more challenging or less predictable than life in captivity [29,30]. Thus, more subtle differences in adrenal function between successful and unsuccessful females may be overwhelmed by the adrenal responses to the more unpredictable wild environment. 

One possible cause of the increase in FGM in unsuccessful pairs might be reproductive suppression. It has been suggested that reproductive suppression among females is likely universal among marmot species [31]. For example, when an older female yellow-bellied marmot is present, a younger female is significantly less likely to wean a litter and it is suggested that this reproductive suppression may also be affected by density. However, of the eleven pairs in whom we assessed FGM during an unsuccessful breeding season, only one female was less than five years of age, so it is unlikely that reproductive suppression by older females housed at the same facility is responsible for the higher FGM in unsuccessful pairs. Reproductive suppression has been reported in male alpine marmots (*Marmota marmota*), with territorial males suppressing reproductive potential in 2-year old juveniles and older non-sons, and this could similarly result in the higher FGM observed in our unsuccessful pairs [32]. But age-related reproductive suppression by older males at the same facility does not appear to be sufficient to explain the higher FGM in unsuccessful pairs for the same reason as the females, since the age distribution of the males in our unsuccessful pairs was fairly even. Furthermore, relative differences in adrenal activity between dominant and subordinate individuals is often context related, with adrenal activity being higher in dominant individuals if their dominance requires repeated effort to maintain [33]. Variables such as dominance or aggression may play a role in the higher FGM in unsuccessful pairs, but only detailed behavioral observations and a more in-depth endocrine analyses would be able to detect this effect. It was also suggested that reproductive suppression may be affected by density, with higher densities making reproductive suppression more likely. However, this is not a likely explanation for the higher FGM we observed in unsuccessful pairs, as the density of adults in the housing facilities was fairly constant from year to year and was similar for successful and unsuccessful pairs. 

Different types of social interactions between the individuals of a breeding pair may provide the best explanation of our endocrine results. Vancouver Island marmots are a social species, living together in colonies made up of at least one or more family groups [3]. The lower FGM concentrations in established marmot pairs, regardless of reproductive success, could be due to social buffering between familiar conspecifics. Social buffering is the moderation of the stress response by the presence of a social partner, with the adrenal response to novelty blunted when social familiars are present [34]. The nature of stress buffering effects has been shown to change by modifying the relative significance of other factors to the buffering effect, such as the familiarity of the buffering animal, with the hormone oxytocin being an important player [35]. Oxytocin increase is associated with the decrease in glucocorticoids observed in social buffering and the administration of exogenous oxytocin has been shown to decrease the glucocorticoid response to separation of social conspecifics [36]. A possible confounding factor is that in six of the eleven new pairs, one or both animals were transferred from another institution, while none of the established pairs changed institutions. Transfers between institutions for breeding take place in the fall prior to hibernation and the breeding season in the following spring for the express purpose of minimizing the effect of stress associated with new housing on breeding success, but this factor can’t be discounted.

Another social interaction more specific to reproduction that could explain the lower FGM concentrations in successful versus unsuccessful marmot pairs was observed, regardless of the initial level of familiarity between the individuals. In the wild, Vancouver Island marmot family groups usually consist of one adult male and one adult female raising their offspring together [3]. This suggests that Vancouver Island marmots are socially monogamous with strong social ties to breeding partners. It is possible that the formation of a pair-bond between opposite-sex individuals is an important aspect of successful breeding in this species. Another monogamous rodent, the prairie vole, is a key species used to understand the physiological and behavioral aspects of pair-bond formation in mammals [37]. In prairie voles, the sexual component of the pair bond consists of a long-term relationship (including co-parenting) in which pair mates prefer to copulate with each other to the point of exclusivity, such that the pair demonstrates a monogamous mating pattern. The hormone oxytocin has been shown to play a critical role in the reinforcing value of the pair-bonded mate relative to other social stimuli [38] and in female prairie voles, cohabitation with a male, leading to partner preference formation, significantly decreased serum corticosterone levels [39]. If the increase in oxytocin associated with pair-bond formation and/or breeding decreases glucocorticoid release like it does during social buffering then this could explain the lower FGM concentrations in both new and established pairs of Vancouver Island marmots that bred successfully. A more detailed investigation measuring both behaviors and endocrine responses in marmot pairs would be required to confirm the relationship between the level of positive social interactions and adrenal activity. Regardless, the observation that the administration of exogenous oxytocin to prairie voles facilitates the formation of pair bonds suggests a possible treatment option to facilitate breeding in the Vancouver Island marmot.

The goal of this study was to determine factors that significantly impacted the odds of successful reproduction in the Vancouver Island marmot, with the idea that the identified variables might be manipulated to improve reproductive success. The manipulation of variables such as parental age, and the duration pairs are housed together, is somewhat constrained when breeding pairs are determined primarily based on genetic relatedness. However, genetic analyses have indicated this approach has indeed been successful at minimizing genetic diversity loss in captive Vancouver Island marmots [40]. Furthermore, managing an endangered species with the goal of maintaining allelic diversity is logical, as there is no crystal ball that wildlife managers can use to determine which alleles will confer an advantage to survival in the wild in the future, so most conservation breeding programs of endangered species take this approach. However, since this approach usually limits mate choice and its associated social value, it may be inadvertently reducing the maximum reproductive potential of the population in human care, particularly in a socially monogamous species like the Vancouver Island marmot where the formation of pair bond may be important to breeding success. Allowing mate-choice has been shown to increase reproductive output in a number of species [41], but the logistics of allowing mate-choice have a number of inherent hurdles, including availability of animals (choices) in very small populations, the risk of intraspecific aggression particularly among males, and a lack of understanding of the parameters involved in choosing a mate in different species. An alternative approach to allowing mate choice in Vancouver Island marmots may be the use of oxytocin administration to facilitate pair-bonds in the pairs dictated by management for genetic diversity.

## 5. Conclusions

Factors associated with the dam have more of an impact on reproductive success in Vancouver Island marmots under human care than factors associated with the sire, but age is a factor for both sexes. Marmots between 5 and 7 years of age have more than double the odds of successfully reproducing in a breeding season than older animals.

Reproduction in the previous year more than doubles the odds of a female marmot successfully reproducing in a breeding season.

Endocrine analyses provided evidence that established and successful pairs of Vancouver Island marmots were less stressed than new or unsuccessful pairs. This is possibly due to the social buffering of the stress response by familiarity and breeding/pair-bond formation. 

The Vancouver Island marmot is critically endangered, which dictates the prioritization of minimizing inbreeding when determining breeding pairs. This may often preclude breeding only animals of certain ages or allowing established pairs to remain together. It is possible that facilitating social bonding between breeding pairs, perhaps by oxytocin administration, would improve reproduction in this socially monogamous species. 

## Figures and Tables

**Table 1:** Univariable analysis of factors influencing reproductive success (weaned litters) in Vancouver Island marmots based on studbook data. Data is presented as odds ratios (OR) and 95% confidence intervals (CI).

Variable	N (%)	OR (95%CI)	*p*-Value
Sire age			0.0059
A (2–4 y)	162 (42.7)	2.122 (1.203–3.743)	
B (5–7 y)	143 (37.7)	2.551 (1.427–4.561)	
C (8–14 y)	74 (19.5)	Reference	
Dam age			0.0114
A (2–4 y)	158 (41.7)	1.400 (0.855–2.294)	
B (5–7 y)	113 (29.8)	2.279 (1.326–3.918)	
C (8–14 y)	108 (28.5)	Reference	
Any previous success–Dam			0.1229
No previous success	163 (43.0)	0.725 (0.481–1.091)	
Previous success	216 (57.0)	Reference	
Success in previous yr–Dam			0.0034
Successful in previous yr	158 (41.7)	1.871 (1.233–2.840)	
Not successful in previous yr	221 (58.3)	Reference	
Any previous success–Sire			0.9939
No previous success	166 (43.8)	0.998 (0.664–1.501)	
Previous success	213 (56.2)	Reference	
Dam transferred			0.1702
Not transferred	297 (78.4)	0.706 (0.429–1.162)	
Transferred	82 (21.6)	Reference	
Sire transferred			0.5579
Not transferred	280 (73.9)	1.147 (0.724–1.818)	
Transferred	99 (26.1)	Reference	
Dam origin			0.2211
Captive	239 (63.1)	0.769 (0.505–1.172)	
Wild	140 (36.9)	Reference	
Sire origin			0.8747
Captive	274 (72.3)	1.037 (0.660–1.629)	
Wild	105 (27.7)	Reference	
Both parents wild			0.8853
Yes	80 (21.1)	1.037 (0.631–1.704)	
No	299 (78.9)	Reference	
Both parents captive			
Yes	214 (56.5)	0.824 (0.547–1.240)	0.3517
No	165 (43.5)	Reference	
Age class difference			0.0702
Parents same age class	204 (53.8)	1.457 (0.969–2.190)	
Parents different age class	175 (46.2)	Reference	
Sire older than dam			0.1765
No	311 (82.1)	1.439 (0.848–2.441)	
Yes	68 (17.9)	Reference	
New pair			0.1509
No	198 (52.2)	1.346 (0.897–2.021)	
Yes	181 (47.8)	Reference	

## Data Availability

Data from the physiological validation of the immunoassay are provided in Figure 1. The endocrine data presented in this study are available on request from the corresponding author. The studbook data are the property of the participating institutions and may be available from Vancouver Island Marmot Recovery Team upon request.
